# The effects of environmental policy uncertainty on productivity growth- Data from Chinese micro enterprise level

**DOI:** 10.1371/journal.pone.0293962

**Published:** 2023-11-08

**Authors:** Dong Le, Fei Ren, Kun Zhang

**Affiliations:** 1 School of Business, Wenzhou University, Wenzhou, Zhejiang, China; 2 School of Economics, Zhongnan University of Economics and Law, Wuhan, Hubei, China; The University of Hong Kong, HONG KONG

## Abstract

China’s economy has made remarkable achievements in the past 40 years. However, the economic development is accompanied by serious environmental pollution. Chinese government has promulgated many policies to reduce environment pollution. However, it is doubtable whether the increased uncertainty in environmental policies inhibits enterprise development. Therefore in this study we use Mathematical Derivation, Stepwise Regression Method and Regulated Effect to investigate the impact of environmental policy uncertainty on enterprise productivity. The results show that (1) environmental policy uncertainty significantly inhibits the improvement of enterprise productivity. (2) environmental policy uncertainty inhibits enterprise productivity by enterprise innovation, human capital and foreign direct investment; (3) environmental policy uncertainty has heterogeneous impact on enterprise productivity. According to this study we also provide some beneficial environmental policy suggestions for the Chinese government. Such as the government should build a stable economic environment, maintain the sustainability of local environmental regulation policies and formulate more detailed measures to adapt different types of enterprises.

## Introduction

In the past 40 years, China’s economy has been growing rapidly, but behind its rapid development, it has faced difficult situations. According to the "China Ecological Environment Status Communique 2022," 218 cities in China meet environmental air quality standards, accounting for only 62.83% of all cities. There are still 126 cities with excessive air pollution emissions, and with just 37.7% of days having decent air quality, air pollution prevention is still in a bad state. The country’s acid rain frequency is 9.4%, and the proportion of cities with acid rain is as high as 33.8%. And the Land pollution is still seriously, the total land area of desertification in the country is 257.37 million square meters, the area of desertification is 168.78 million square meters, and the land size of the existing rocky desertification region in the karst area is 723,000 hectares. A large number of epidemiological and toxicological studies have shown that environmental pollution such as air pollution, soil pollution, and water pollution can lead to chronic poisoning of the human body, as well as increase the risk of human cancer. Serious environmental pollution not only impedes China’s economy’s high-quality development, but also poses a significant threat to citizens’ health. Therefore the government has issued some policies to strengthen the protection of environment, Air Pollution Prevention Law of the People’s Republic of China (2014), Water Pollution Prevention Law of the People’s Republic of China (2018), Law of the People’s Republic of China on the Prevention and Control of Air Pollution (2021) and so on, which has undoubtedly effectively alleviated the enterprises’ pollution discharge.

At present, EPU (environmental policy uncertainty) is a typical feature of China. Since the central government proposed strengthening the construction of socialist ecological civilisation, the government has successively issued the Environmental Protection Law (2014), revised Atmospheric Pollution Prevention Law (2015), and issued Soil Pollution Prevention and Control Action Plan (2016). The promulgation of these policies has effectively alleviated the serious pollution discharge of enterprises and degradation of the ecosystem, but the negative effects of increased EPU are becoming more apparent. Since enterprise often face a significant amount of uncertainty regarding the timing, content, and potential impact of environment policy decisions, and the business decisions of enterprises are largely reliant on the government’s environment policies. To this effect, EPU is an important risk for business activities [1]. Meanwhile, the increase in EPU caused by policy changes also impacts enterprise production and investment income, it had led enterprises to be particularly cautious and remain reluctant to hire or expand capacity [2]. Bloom’s (2009) [3] study also shows that EPU may lead to reduce productivity growth and pollution deterioration. Even though there are many commentators argue that EPU is one of the main reasons for the EP’s sluggish increase (enterprise productivity), and this problem has been studied from a macro point of view. But previous studies have not systematized and modeled this problem, and this paper will construct a model to systematically study this problem. In this study we contribute to this debate by mathematical derivation and empirical analysis from Chinese micro enterprise level. This can provide a basis for the government to formulate relevant policies, which will be conducive to the development of enterprises.

### Literature review

#### Visual analysis of literature

Based on the Web of Science paper library, this study conducts an in-depth analysis of the literature characteristics and trends of EPU. This study analyses the hot trends of EPU through co-word analysis, cluster analysis, and strategic coordinate analysis. On coword analysis and cluster analysis, strategic coordinate analysis shows the content change and development trend of the research topic intuitively through two-dimensional plane coordinate.

#### Overall growth trend analysis

**Fig 1:**
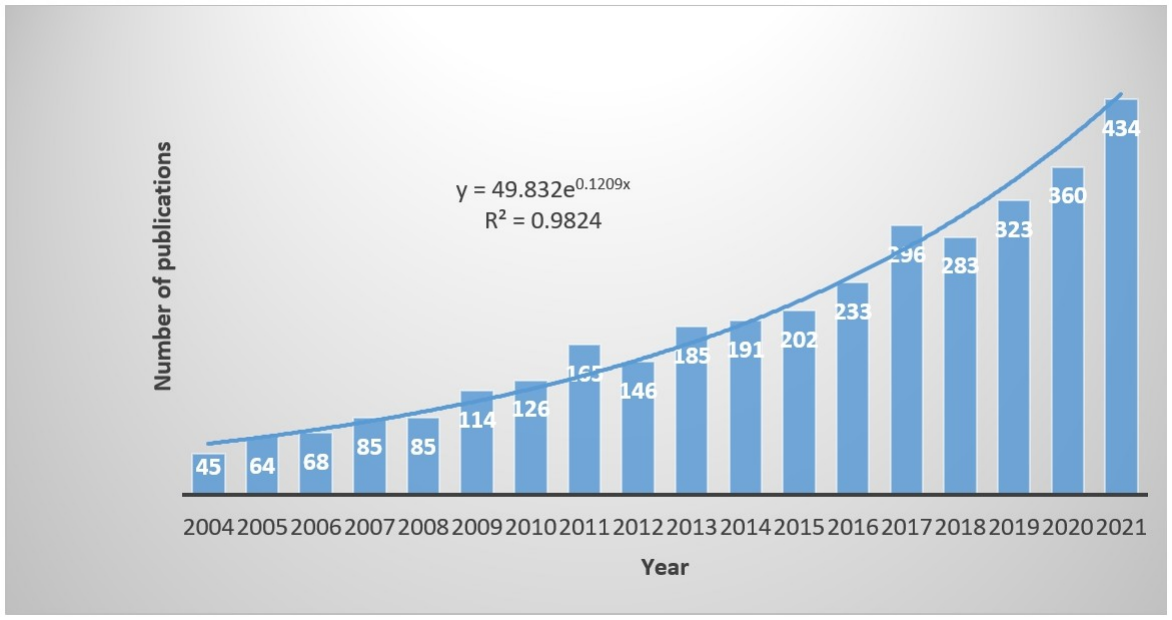
Number of EPU published in past years.

The number of EPU published in the past years from 2004 to 2021 based on the WOS core paper database (Fig 1) has been increasing year by year and has received increasing attention in academic circles.

#### Keywords co-word visual analysis

**Fig 2:**
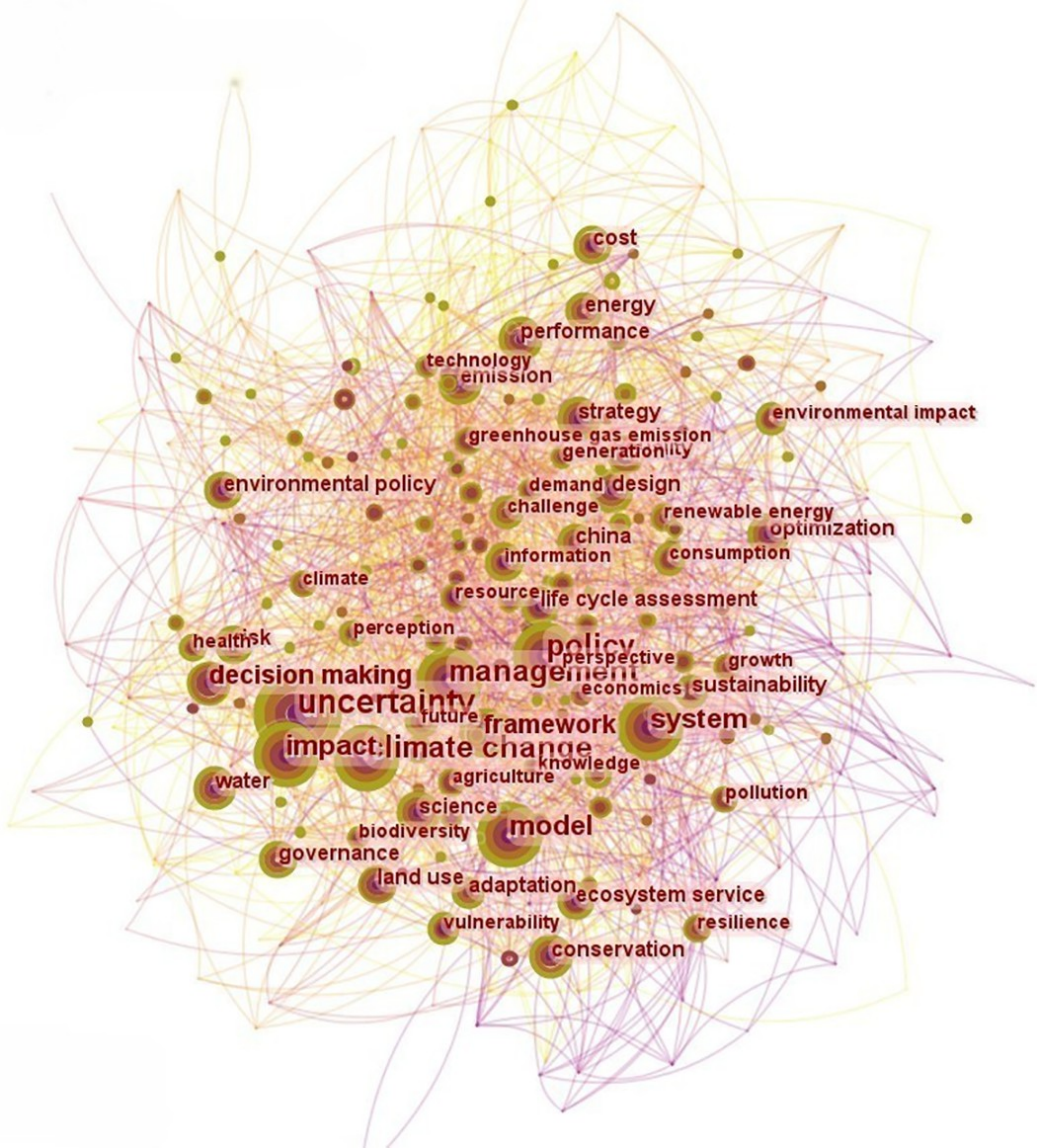
Co-occurrence picture of EPU from 2000 to 2021. Note: The figure is similar but not identical to the original image and is therefore for illustrative purposes only.

The visualization function of Citespace can conduct in-depth analysis of keywords in many literatures, and further understand the hot spots of EPU in economic research by interpreting the co-occurrence map of keywords. It can be observed that the node size is positively correlated with the frequency of keyword occurrence. Uncertainty, policy, decision-making, system, economics, management, performance, and environmental impact were the most frequently used keywords in the study of EPU from 2000 to 2021 (Fig 2).

#### Strategic coordinate analysis

**Fig 3:**
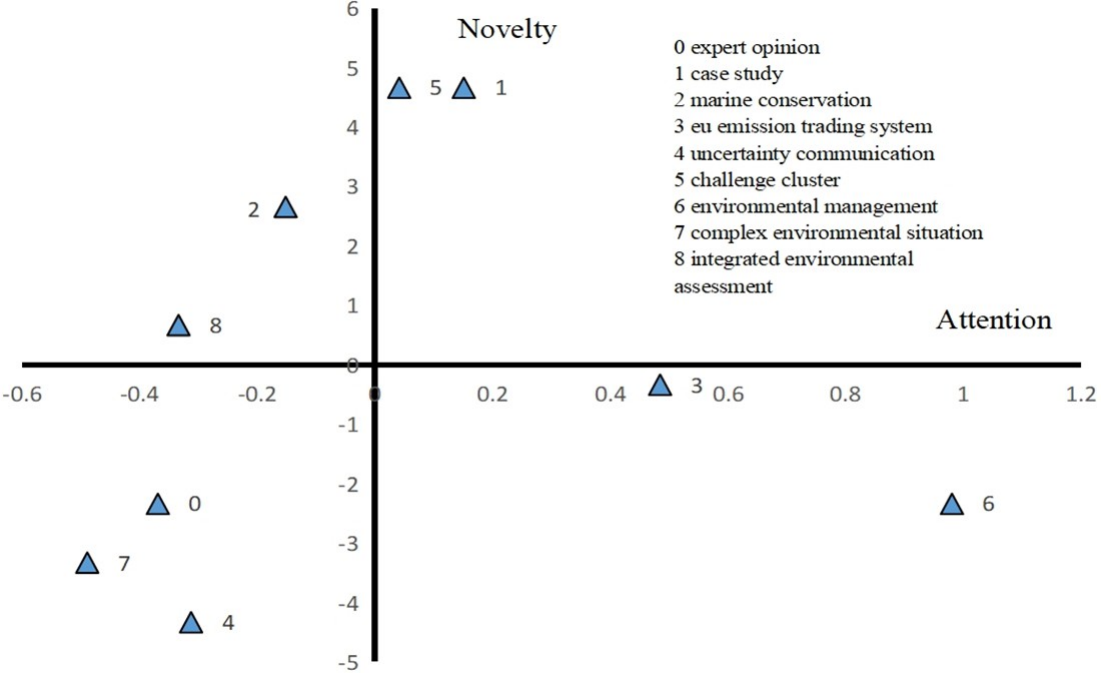
Strategic coordinates of EPU 2000–2021.

Strategic coordinates are mainly used to analyse the structure and change trend of a research field. In this study, we construct indicators of attention and novelty degree. By constructing attention and novelty indicators, we drew a strategic coordinate map of EPU from 2000 to 2021 (Fig 3). The “core domain” in the first quadrant is the hot spot of EPU research from 2000 to 2021, and cluster 6 has the highest attention, indicating that environmental management receives the highest attention and is also a hot topic for scholars to study. Cluster 5 and cluster 1 belong to the research field with the highest novelty degree, indicating that “Case study” and “Challenge cluster” are the research hotspots in recent years. Clusters 2 and 8 in the second quadrant belong to "potential fields", indicating that although the research content of these clusters is not widely concerned, it may become the core content of EPU research in the future. In particular, cluster 2 "Marine conservation" has a high novelty level. However, clusters 0, 4 and 7 in the third quadrant of the "marginal field", the novelty degree and attention are negative, indicating that they are marginalized in the field of EPU. In the fourth quadrant, clusters 3 and 6 receive more attention than 0, but their novelty degree is less than 0. Based on the novelty degree, these clusters are not new research hotspots. However, these topics have been of concern for a long time and have been the most academic literature in the field of EPU research.

### Literature summary

#### Enterprise productivity (EP)

EP reflects the efficiency of converting enterprise inputs into output costs [4]. Research on enterprise production mainly focuses on measurement methods and influencing factors, such as OP, LP, and GMM, and parametric methods, such as DEA, to measure EP. The semi-parametric method OP mainly makes investments according to the productivity status of the enterprise, and it takes the current investment as the proxy variable of EP to solve the problem of simultaneous bias in the estimation process. Levinsohn and Petrin (2003) used a wide range of intermediate products as proxy variables, enabling the LP method to measure EP based on data availability [5]. Blundell and Bond (1998) use GMM to solve the problems of endogeneity and simultaneity bias in the model [6]. The parameter method DEA model does not need to set a fixed function form, which reduces subjective factors. Most importantly, when the DEA has multiple decision units, it can be compared with the optimal decision unit to obtain the corresponding efficiency value of each decision unit. When using DEA to measure efficiency, Tone measured input-output efficiency from a non-radial angle (DEA-SBM), avoiding measurement errors in the radial angle.

As for the influencing factors of EP, scholars mainly carry out relevant studies from the aspects of technological innovation, human capital, and foreign investment. Schumpeter (1934) proposed that technological innovation breaks resource constraints by promoting the reorganisation of production [7]. Technological innovation can promote the reorganisation of production factors, and thus improve EP [8]. Rosenbusch et al. (2011) used meta-analysis to conclude that technological innovation can promote EP [9]. When Baumann and Kritikos (2016) studied the data of small- and medium-sized enterprises in Germany, they found that innovation in small- and medium-sized enterprises can also promote EP, which is not significantly different from large enterprises [10].

With globalisation of the industrial division of labour, human capital has gradually become an important factor in improving EP. Moretti (2004) found that human capital had a significant positive correlation with EP through the data of urban human capital and micro-EP [11]. Subramaniam and Youndt (2005) found that human capital, social capital, and their relationships can significantly promote EP [12]. Rice et al. (2006) used the NUTS3 data of the United Kingdom to find that the higher the proportion of workers with higher degrees, the more obvious is the promotion effect on EP [13]. Oliver (2015) used years of education to measure human capital and found that the more years of education an employee has, the more significant is the promotion effect on EP [14]. Teixeira and Queirós (2016) proposed that human capital mainly promotes EP by improving workers’ skills and learning abilities [15].

Since the beginning of the new century, China has further improved the degree of opening up, and foreign direct investment has become an indispensable part of promoting the development of enterprises. The intensification of economic globalization leads to the spillover effect of investment [16]. Liu et al. (2000) found that the productivity of manufacturing enterprises is directly proportional to foreign investment by analysing data from the British manufacturing industry [17]. Ke (2010) proposed that FDI can improve the capital and industrial structures of local enterprises and increase the foreign exchange reserves of host countries to promote EP [18]. Hamida (2013) found that the Swiss manufacturing industry can absorb advanced technological experience from the positive spillover effect of foreign direct investment to promote its own EP [19]. According to Fujimori and Sato’s research, increasing foreign investment in firms will greatly boost EP, and the long-term favourable link between foreign direct investment and EP will remain [20].

#### EPU

The existing literature on EPU mainly focuses on its connotation and impact on enterprise development. Uncertainty refers to the behaviour subject must make a corresponding decision when it cannot grasp all the information. Uncertainty is one of the factors affecting investment return [21]. When the object of uncertainty is environmental policy, EPU is formed [22].

Policy uncertainty affects firms’ cash flows, financial constraints, and risk channels [23]. The cost of enterprise decision-making will be increased when the environmental policies are changing dynamically, it is also not conducive to the transformation of enterprise innovation achievements [24]. The influx of foreign capital causes China’s enterprises to face an increased degree of uncertainty [25]. Furthermore, the EPU makes it harder for businesses to make accurate predictions about the market environment, raises the risk of financial markets, and forces businesses to cut investment in technology innovation and human resources [26], this further impedes EP progress. And EPU has a negative influence on EP mostly through its effects on the capital condition of enterprises [27]. According to Nithya’s research, EPU has a detrimental impact on the firm’s productivity increase [28]. Ng et al (2023) assess the influence of EPU on sustainability performance using country-level annual data, and they suggest that EPU could have major ramifications for local economies, hence impeding sustainability progress [29].

### Summary

Throughout the existing literature, scholars have carried out relevant studies on the measurement methods and influencing factors of EP, and have also carried out relevant analysis from the connotation of EPU and its impact on foreign investment, residents’ employment, and other aspects. However, whether EPU has an impact on EP is seldom mentioned. Owing to the lack of an effective micro basis for research at the macro level, it is not clear how the influence mechanism at the macro level acts on the productivity of micro enterprises. In fact, dealing with an increase in EPU is important to understanding the exact relationship between macro-level policy fluctuations and EP. Therefore, based on combined data from the China Industrial Enterprise Database(CIED), China Patent Database(CPD), and China Industrial Enterprise Pollution Emission Database(CIEPED) from 2005 to 2014, this study illustrates the impact and mechanism of EPU on EP from the perspective of micro enterprises.

## Theoretical support and model

### Theoretical analysis

#### Demand and preference

Mathematical Derivation often separates all components other than the subject of inquiry from the model and keeps only some significant traits while ignoring all other features, examining and researching in the purest sense. Meanwhile Mathematical Derivations are based on the relationship between EPU and EP in this study, mathematical models are formed by solving equations, and practical problems are solved based on the results. So based on the theoretical model of Handley (2017) [30], appropriate adjustments were made according to the research purpose to analyse the impact of EPU on EP. Assume that consumers have a risk aversion (CRRA) preference:

$$U_{0}=\int_{0}^{\infty }\text{exp}(-\alpha t)\frac{C{\left(t\right)}^{1-\chi }-1}{1-\chi }dt$$ (1)

*α* > 0 is the discount factor and *χ* > 0, *χ* ≠ 1 is the relative risk aversion factor, Then total consumption *C*(*t*) can be expressed as:

$$C(t)={\left[\int c_{j}{\left(t\right)}^{\frac{\delta -1}{\delta }}dj\right]}^{\frac{\delta }{\delta -1}}$$ (2)

where *c*_*j*_(*t*) represents the consumption of product *j* at *t δ* > 1 is the elasticity of product substitution.

#### R&D and production

R&D investment expense *G*_*i*,*j*_ is the sunk cost of enterprise *i* entering the market. We assume that the production rate of product *j* produced by the monopolistic competitor is 1/*c*_*j*_. Then, the production function of enterprise A is

$$y_{i,j}=A_{i,j}l_{i,j}k_{i}$$ (3)

*A*_*i*,*j*_ and *l*_*i*,*j*_ are the amount of technology and labour used by enterprise *i* to produce *j*, respectively. Then, the above equation can be simplified as

$$y_{i,j}={\hat{A}}_{i,j}l_{i,j}$$ (4)

${\hat{A}}_{i,j}=\frac{y_{i,j}}{l_{i,j}}$ is the marginal output of labour input, that is, labour productivity. The marginal cost of product *j* is $\frac{\omega }{\hat{A}}$. When the wage is price rigid, the equilibrium wage *ω* is not affected by uncertainty.

#### Price and profit

The inverse demand function of the total consumption *C*(*t*) is $p_{j}=C^{\frac{1}{\delta }}c_{j}^{-\frac{1}{\delta }}$. Suppose the firm obtains the $\frac{p_{j}}{\lambda }$ profit per unit of output; *λ* ≥ 1 is the degree of uncertainty. Then, the profit maximisation function of the manufacturer is

$$\pi ({\hat{A}}_{j})={\text{max}}_{c_{j}\geq 0}\left\{(C^{\frac{1}{\delta }}c_{j}^{-\frac{1}{\delta }}/\lambda -\omega_{j}{\hat{A}}_{{}_{j}}^{-1})c_{j}\right\}$$ (5)

then:

$$p_{j}=(\frac{\delta }{\delta -1})\lambda \omega_{j}{\hat{A}}_{{}_{j}}^{-1}$$ (6)

$$c_{j}={\left(\frac{\delta -1}{\delta }\right)}^{\delta }\lambda^{-\delta }C\omega_{{}_{j}}^{-\delta }{\hat{A}}_{{}_{j}}^{\delta }$$ (7)

Equilibrium profit is:

$$\pi =\xi \omega_{{}_{j}}^{1-\delta }{\hat{A}}_{{}_{j}}^{\delta -1}$$ (8)

and *ξ* = (*λδ*)^−*δ*^ (*δ* − 1)^*δ*−1^.

#### Policy uncertainties and general equilibrium

The exogenous policy process encompasses three policy critical states: b = 0,1,2, and *λ*_2_ > *λ*_0_. Intermediate state *λ*_1_ ∈ (*λ*_0_, *λ*_2_). It varies with the probability of *φ* > 0. The policy system is characterised by a Markov process of constant distribution called ^(*λ*_*b*_, *φ*). The policy change matrix is:

$$B=\left[\begin{array}{ccc} \eta_{22} & \eta_{21} & 0\\ \eta_{12} & \eta_{11}=1-\varphi & \eta_{10}\\ 0 & 0 & 1 \end{array}\right]$$ (9)

When the enterprise enters the market, the state of the mall is *m*, and the current economic situation is *e*_*m*_. The expected value of an enterprise after entering the market is:

$$\prod_{w}(e_{m},\hat{A},\varphi )=\pi (e_{m},{\hat{A}}_{j},\varphi )+\beta W_{m}\prod_{w}(e_{m}^{\prime },\hat{A},\varphi )$$ (10)

where *W*_*m*_ represents the future expectation based on the current state information set *m*, and *β* < 1 is the probability of the enterprise entering the market and continuing production in the future period. The baseline thresholds are as follows:

$$\frac{\pi (e_{m},{\hat{A}}^{b},\varphi )}{1-\beta }=G\Leftrightarrow {\hat{A}}_{m}^{b}=\omega_{m}^{-1}{\left[\frac{(1-\beta )G}{e_{m}}\right]}^{\frac{1}{\delta -1}}$$ (11)

The optimal entry decision that maximises the expected value of the enterprise in state m is

$$\prod (e_{m},\hat{A},\varphi )=\text{max}\left\{\prod_{w}(e_{m},\hat{A},\varphi )-G,\beta W_{m}\prod (e_{m}^{\prime },\hat{A},\varphi )\right\}$$ (12)

Can be converted to:

$$\prod (e_{m},\hat{A},\varphi )-\prod_{w}(e_{m},\hat{A},\varphi )+G=\text{max}\left\{0,\beta W_{m}\left[\prod (e_{m}^{\prime },\hat{A},\varphi )-\prod_{w}(e_{m}^{\prime },\hat{A},\varphi )\right]-\pi (e_{m},\hat{A},\varphi )+G\right\}$$ (13)

then

$$X_{m}=\text{max}\left\{0,\beta W_{m}X_{m}^{\prime }-\pi (e_{m},\hat{A},\varphi )-G(1-\beta )\right\}$$ (14)

The value of deferred option is:

$$X_{m}\equiv \prod (e_{m},\hat{A},\varphi )-\prod_{w}(e_{m},\hat{A},\varphi )+G$$ (15)

$$W_{m}X_{m}^{\prime }=W_{m}\left[\prod (e_{m}^{\prime },\hat{A},\varphi )-\prod_{w}(e_{m}^{\prime },\hat{A},\varphi )+G\right]$$ (16)

Combined with Formula (14), it can be obtained:

$$\begin{array}{l} W_{m}X_{m}^{\prime }=\eta_{m,m+1}\left[\beta W_{m+1}X_{m}^{\prime }-\pi (e_{m+1},\hat{A})+G(1-\beta )\right]\\ =\frac{\eta_{m,m+1}}{1-\beta \eta_{m+1,m+1}}\left[G(1-\beta )-\pi (e_{m+1},\hat{A})\right] \end{array}$$ (17)

The expectation of the enterprise in m+1 state is:

$$\begin{array}{l} W_{m+1}X_{m}^{\prime }=\eta_{m+1,m+1}\left[\beta W_{m+1}X_{m}^{\prime }-\pi (e_{m+1},\hat{A})+G(1-\beta )\right]\\ =\frac{\eta_{m+1,m+1}}{1-\beta \eta_{m+1,m+1}}\left[G(1-\beta )-\pi (e_{m+1},\hat{A})\right] \end{array}$$ (18)

According to definitions of $\pi (e_{m},{\hat{A}}_{{}_{m}}^{b},\varphi )=G(1-\beta )$ and, $X_{m}({\hat{A}}_{m}^{b})=\text{max}\left\{0,\beta W_{m}X_{m}^{\prime }({\hat{A}}_{m}^{b})\right\}$, we can see that the enterprise has a non-negative delay option value under uncertain conditions, namely, $X_{m}({\hat{A}}_{m}^{b})\geq 0$, according to Formula (14):

$$\beta W_{m}X_{m}^{\prime }-\pi (e_{m},{\hat{A}}_{m}^{b},\varphi )+G(1-\beta )\geq 0$$ (19)

get: $W_{m}X_{m}^{\prime }\geq 0$, so: $\pi (e_{m+1},\hat{A})\leq G(1-\beta )=\pi (e_{m+1},{\hat{A}}_{m}^{b},\varphi )$ can be obtained according to formula (8): ${\hat{A}}_{m}^{b}\geq {\hat{A}}_{m}^{r}$, Therefore, EP decreases under uncertain conditions.

### Mechanism analysis

The improvement of enterprise innovation, stock of human capital and increase of enterprise investment have become the key factors affecting the long-term economic growth [31]. Therefore, this section mainly analyses the impact of EPU on EP from three aspects: enterprise innovation, human capital and foreign direct investment.

#### EPU inhibits the growth of EP by hindering the improvement of enterprise innovation

In order to realize the green development of enterprises, the government has issued some regulation policies to reduce enterprises’ emission. However, the frequent fluctuation of regulation policies leads to the increase of EPU, which has a certain negative impact on EP. Technological innovation can bring monopoly profits to enterprises [32], but it also needs to invest a large amount of capital, labor and other production factors. In addition, inter-enterprise technological cooperation and labor flow will also lead to technology leakage. These make the enterprise technology investment income uncertainty increase [33]. When faced with the increase of EPU, enterprises will retain sufficient funds to cope with the unknown impact. Meanwhile, the increase of EPU further increases the difficulty of enterprise financing. Therefore, in order to avoid risks, enterprises will increase short-term investment with clear investment returns, then the innovation investment of enterprises is insufficient, which further inhibits enterprises’ innovation. The inhibition of enterprise innovation ability by EPU will make enterprises unable to obtain advanced green production technology and face the dilemma of low energy utilization efficiency, also failure to break through resource and environmental constraints [34], which will eventually hinder the growth of EP.

#### EPU inhibits the growth of EP by hindering the improvement of human capital stock

When EPU increases, enterprise tend to make short-term investments with clear benefits. However, human capital input has long-term and low returns, and the adjustment cost of human capital is low compared to other factors of production [35]. And there may be gender differences [36]. Enterprise investment in human capital needs to be based on basic market information such as sales budget, input, and output; EPU makes it difficult for enterprise managers to make accurate assessment of market changes. Then enterprises tend to keep more cash to resist enterprises may face the unknown risks; therefore, when the uncertainty of environmental policy increases, enterprises will reduce their investment in human capital [37]. However, human capital promotes innovation in energy-saving technology and maximises the resource utilisation efficiency of products, thus promoting the improvement of EP. Therefore, EPU inhibits the growth of EP by hindering the improvement of human capital stock.

#### EPU inhibits the growth of EP by hindering foreign direct investment(FDI)

As multinational investment enterprises have abundant funds, with low pollution emission and high level of green production technology. Therefore, attracting foreign capital has become one of the main channels for China to introduce green technology. And foreign enterprises will enhance the competitiveness of the domestic market, so as to force local enterprises to introduce advanced production technology, reduce production costs to improve product competitiveness. When China’s EPU increases, in order to avoid risks and maximize profits, foreign investor tend to choose investment with clear and stable returns, or even choose not to invest in order to reduce the risk of investment loss, thus they will reduce investment in China. Uncertainty can affect a firm’s financing constraints [38]. FDI is an important way for developing countries to connect with foreign markets, obtain technology spillover, improve production capacity and expand employment scale [39]. The reduced scale of foreign direct investment is not conducive to the introduction of green technology by Chinese domestic enterprises, but also makes it impossible for enterprises to obtain the production scale effect brought by foreign investment. Therefore, EPU hinders the improvement of EP by inhibiting FDI.

### Models, variables, and data

#### Model specification

To verify the impact of EPU on EP, the benchmark model is set as follows:

$$ep_{ijkt}=\beta_{0}+\beta_{1}epu_{ijkt}+\beta_{2}X_{ijkt}+\lambda_{i}+\lambda_{j}+\lambda_{k}+\lambda_{t}+\varepsilon_{ijkt}$$ (20)

Subscript *i*, *j*, *k*, *t* indicates the enterprise, industry, region, and year respectively, the explained variable *ep* represents EP. Core explanatory variable *epu* represents EPU. *X*_*ijkt*_ is the control variable, *λ*_*i*_, *λ*_*j*_, *λ*_*k*_, *λ*_*t*_ represents firm, industry, region, and year fixed effect respectively. *ε*_*ijkt*_ is the random perturbation term.

#### Variable selection

*Explained variable EP*. This study used the data of CIED from 2005 to 2014 to calculate EP. This database is the most authoritative enterprise information database in China, and mainly contains the basic information of all public and private enterprises. The number of enterprises counted every year is up to 300,000, with large sample size and comprehensive basic information. The semi-parametric method LP is used to measure EP, and the robustness test is carried out by using OP method.

*Explanatory variables EPU*. The increase of EPU will lead to the change of enterprise sales revenue [40]. Therefore, EPU can be measured by fluctuations in company sales revenue [41]. The standard difference in sales income can be used to measure the uncertainty index of environmental policy, whereas Ghosh and Olsen (2009) used the standard deviation of company sales revenue to measure EPU [42]. OLS was used to estimate the abnormal sales revenue of enterprises over the past three years:

$$Sale=\varphi_{0}+\varphi_{1}Year+\varepsilon$$ (21)

where *Sale* is the sales revenue of the enterprise and *Year* is the year. Formula (21) Regression residual is the abnormal sales income of the enterprise and calculates the standard deviation of abnormal sales income of the enterprise in the past three years, dividing the calculated standard deviation by the average sales income of the enterprise in the past three years, to obtain the EPU without industry adjustment. EPU without industry adjustment is divided by the median of EPU to eliminate the influence of industry bias and finally obtain the EPU without industry bias (*epu*).

*Moderating variables*. The moderating variable, enterprise innovation *iq*, was measured by the total number of patent applications and grants annually. Existing literature believes that employees without higher education are usually not within the scope of human capital statistics [43]. Therefore, the proportion of employees with higher education was selected to measure the human capital of an enterprise (*rlzb*). The amount of foreign direct investment in local enterprises (100 million) was selected to measure foreign direct investment (*fdi*).

*Other variables*. The selection of control variables mainly referred to the practice of Jung et al. (2014) [35]. The selection mainly includes industrial structure (*is*), which is measured by the ratio of regional secondary industrial output value to GDP. Enterprise fixed assets (*gdzc*): Measured by the total value of enterprise fixed assets (10 million). Pollution emission intensity (*wrpfqd*): measured as the ratio of SO_2_ emissions to the total output value of an enterprise. Asset-liability ratio (*zcfzl*): measured as the ratio of total corporate liabilities to total corporate assets. Dummy variable for state-owned enterprises (*sfgy*):1 for absolute state-owned holdings and 0 for other cases (*hyfl*): Industry category 0 represents the polluting industry and 1 represents the clean industry. Enterprise size (*qygm*): If the number of employees exceeds 1000, the value is 1 for large enterprises and 0 for small and medium enterprises; waste management intensity (*fwzlqd*): measured by the sum of the enterprise’s wastewater and exhaust treatment facilities.

#### Data source and processing

The data source of this study is a combination of data from the China Industrial Enterprise Database, China Industrial Enterprise Pollution Emission Database, and China Patent Database. The China Industrial Enterprise Database contains information on all state-owned and non-state-owned enterprises with an annual output value of more than 5 million yuan in China, mainly including enterprise name, organisation code, legal representative, holding status, main business income, and other information. The pollution emission database of China’s industrial enterprises contain the most original data collected by the National Bureau of Statistics on the pollution emissions of industrial enterprises. The content in the table mainly includes the enterprise name, legal representative, organisation code, wastewater, exhaust gas, and other pollution emission indicators. First, the two types of data are combined and processed according to commonly used processing methods (Brandt et al., 2014) [44]. The State Intellectual Property Office of the People’s Republic of China is the authoritative source of patent databases in China. It mainly statistics the patent applications and authorisation of enterprises over the years. The content of Patent database in China mainly includes enterprise name, organization code, invention patent, utility model patent and design patent and other basic information. The combined data of China Patent Database, China industrial Enterprise database and China industrial enterprise pollution emission database were also combined through the above matching method. Finally, approximately 197000 enterprises participated in the analysis.

Since the latest statistical year of CIED is 2014, this study uses combined data from 2005 to 2014 to conduct regression. According to the descriptive statistics for the variables (Table 1), there is a large difference in EP, with the minimum value being 1.034 and the maximum value being 11.940. There is also a large gap in the EPU faced by enterprises, with a minimum value of 0.224 and a maximum value of 11.950.

**Table 1:** Descriptive statistics of the variables.

Variables	Mean	Std. Dev	Min	Max
ep	7.046	1.749	1.034	11.940
epu	1.527	1.658	0.224	11.950
is	0.499	0.053	0.213	0.590
gdzc	1.523	9.725	0.001	980.000
wrpfqd	9.317	29.590	0.001	288.700
zcfzl	0.571	0.310	-0.541	19.810

Note: This table reports the variable’s symbol, mean, standard deviation (Std. Dev.), minimum (Min.), maximum (Max.), median.

## Results

### Interpretations of results

The primary principle behind stepwise regression is to lower the degree of multicollinearity by removing less important variables that are highly associated with other variables. Variables are introduced into the model one by one, and the selected explanatory variables are tested one by one. This is an iterative process until no significant explanatory variables are included in the regression equation, and no insignificant explanatory variables are removed from the regression equation, so as to ensure that the final set of explanatory variables is optimal. The stepwise regression method has the advantage of eliminating statistically unimportant explanatory factors, hiding multicollinearity amongst explanatory variables retained in the model, and providing a stronger explanatory contribution to the explained variables. So we choose stepwise regression to analyse the effects of EPU on EP. The correlation between EPU and EP is significantly negative (Table 2), indicating that EPU significantly inhibits the improvement of EP. When faced with the increase of EPU, enterprises will retain sufficient funds to cope with the unknown impact. Meanwhile, the increase of EPU further increases the difficulty of enterprise financing. Therefore, in order to avoid risks, enterprises will increase short-term investment with clear investment returns, then the innovation investment of enterprises is insufficient, which further inhibits enterprises’ innovation.

**Table 2:** Stepwise regression results.

Variables	(1)	(2)	(3)	(4)	(5)
	ep	ep	ep	ep	ep
epu	-0.011***	-0.011***	-0.011***	-0.006**	-0.041***
	(0.003)	(0.003)	(0.003)	(0.003)	(0.008)
is		1.968***	2.001***	1.585**	1.452*
		(0.631)	(0.631)	(0.729)	(0.849)
gdzc			0.009***	0.010***	0.014***
			(0.003)	(0.003)	(0.004)
wrpfqd				-0.004***	-0.003**
				(0.001)	(0.001)
zcfzl					-0.085
					(0.123)
Enterprise	Yes	Yes	Yes	Yes	Yes
Industry	Yes	Yes	Yes	Yes	Yes
Region	Yes	Yes	Yes	Yes	Yes
Time	Yes	Yes	Yes	Yes	Yes
Obs	23,268	22,866	22,866	17,739	16,548
R-squared	0.055	0.296	0.297	0.315	0.432

Note: Z-statistics values are reported in parentheses();

*, **, *** denote the significance at 10%, 5%, 1% levels, respectively.

Enterprise investment in human capital needs to be based on basic market information such as sales budget, input, and output. EPU makes it difficult for enterprise managers to make accurate assessment of market changes. Then enterprises tend to keep more cash to resist enterprises may face the unknown risks, therefore, when the uncertainty of environmental policy increases, enterprises will reduce their investment in human capital [45].

Policy uncertainty affects investment returns [46]. When China’s EPU increases, in order to maximize profits, foreign investor tend to choose investment with clear and stable returns, thus they will reduce investment in China. From the perspective of reducing risks, foreign investors prefer enterprises with clear and stable investment returns, Therefore, the increase of EPU reduces foreign direct investment, the reduction of foreign investment further reduces the financial and operational risks faced by enterprises, further blocks the overflow channels of advanced technology and talents from developed countries, and makes the innovation ability, human capital stock and foreign investment of enterprises at a low level [47]. So, when China’s EPU rises, it considerably impedes the improvement of enterprise innovation, the improvement of human resource stock, foreign direct investment (FDI), and, finally, the increase of EPU significantly hinders the improvement of EP.

### Heterogeneity analysis

#### Heterogeneity of enterprise ownership

Enterprises can be divided into state-owned enterprises and non-state-owned enterprises according to their shareholding. The business performance of enterprises is often heterogeneous due to the nature of enterprises [48]. In state-owned enterprises, EPU has no significant impact on EP, while in non-state-owned enterprises, EPU has a greater impact on EP (Table 3). As state-owned enterprises are controlled by the government, their capital is relatively abundant and their returns are clearer than those of private enterprises. Consequently, state-owned enterprises are more attractive to talent and foreign capital, their human capital stock is higher, and their technology is more advanced. Therefore, when environmental policies fluctuate, they are less negatively impacted by EPU. Non-state-owned enterprises are mostly private enterprises and mixed-ownership enterprises, which have limited capital scale, high financing difficulty, and low ability to attract talent. Consequently, private enterprises have a low stock of human capital, poor technical levels, and low comprehensive quality of managers [49]. Therefore, when the uncertainty of environmental policy increases, it has a significant impact on non-state-owned enterprises. Thus, EPU significantly inhibits the productivity improvement of non-state-owned enterprises.

**Table 3:** Heterogeneity analysis of enterprise ownership and industry categories.

Variables	State-owned enterprises	Non-state-owned enterprise	Cleaning industry	Polluting industries
	ep	ep	ep	ep
ep	-0.031	-0.041***	-0.010	-0.074***
	(0.042)	(0.009)	(0.015)	(0.012)
Control variables	Yes	Yes	Yes	Yes
Enterprise	Yes	Yes	Yes	Yes
Industry	Yes	Yes	Yes	Yes
Region	Yes	Yes	Yes	Yes
Time	Yes	Yes	Yes	Yes
Obs	1,767	14,815	4,012	12,888
R-squared	0.586	0.421	0.510	0.440

Note: Z-statistics values are reported in parentheses();

*, **, *** denote the significance at 10%, 5%, 1% levels, respectively.

#### Enterprise industry category heterogeneity

The impact of EPU on EP in the clean industry does not pass the significance test, but the impact of EPU on EP in the polluting industry is significantly negative (Table 3). Since most cleaning industry enterprises’ pollution emission intensity is low. Therefore, EPU has a low impact on the productivity of cleaning industry enterprises. However, enterprises in polluting industries have a high pollution emission intensity. When the government’s environmental regulation intensity is low, enterprises emit more pollution. When the government’s environmental regulation intensity is high, enterprises reduce their pollution emissions, which increases their investment in enterprise emission reduction and leads to a substantial increase in the production cost of enterprises [50]. Therefore, when EPU increases, the productivity of enterprises in polluting industries decreases.

#### Enterprise age heterogeneity

Enterprises are divided into young and elderly enterprises according to their median age. The negative effect of EPU on the productivity of young enterprises is greater than that of elderly enterprises (Table 4). Young enterprises have been established for a short time, lack sufficient funds to introduce advanced production equipment and management talent in the development stage, and it is difficult for enterprises to obtain foreign investment. The increasing uncertainty of environmental policy causes young enterprises to keep more cash to prevent adverse consequences in the future. Investment in innovation and talent training has decreased, while older enterprises have been established for a long time and have a certain production experience and advanced equipment to cope with environmental policy uncertainties. Compared with younger enterprises, older enterprises are in a stable development period, and their financing ability, innovation ability, and sewage equipment are better than those of younger enterprises in the initial stage [51]. Therefore, EPU has a greater negative impact on the productivity of young firms than on old ones.

**Table 4:** Heterogeneity analysis of firm age and firm size.

Variables	Young enterprises	Elderly enterprises	Large enterprises	Small and medium-sized enterprises
	ep	ep	ep	ep
epu	-0.106***	-0.025***	-0.051	-0.040***
	(0.019)	(0.009)	(0.031)	(0.009)
Control variables	Yes	Yes	Yes	Yes
Enterprise	Yes	Yes	Yes	Yes
Industry	Yes	Yes	Yes	Yes
Region	Yes	Yes	Yes	Yes
Time	Yes	Yes	Yes	Yes
Obs	8,567	11,644	2,396	14,301
R-squared	0.456	0.443	0.609	0.396

Note: Z-statistics values are reported in parentheses();

*, **, *** denote the significance at 10%, 5%, 1% levels, respectively.

#### Heterogeneity of firm size

Enterprises are divided into large enterprises and small- and medium-sized enterprises. Large enterprises are better than small and medium-sized enterprises in terms of enterprise scale, sewage technology and equipment, and financing capacity. Therefore, when EPU increases, large enterprises have sufficient capital, advanced production equipment and management personnel to cope with shocks. Therefore, in large enterprises, EPU has a less negative impact on EP. Small and medium-sized enterprises are inferior to large enterprises in terms of scale, capital reserves, production equipment, and management personnel. When facing EPU, they are forced to reduce investment so they can reserve sufficient funds to face the possible impact. Therefore, EPU has a greater negative impact on the productivity of SMES than large enterprises.

#### Enterprise type heterogeneity

Enterprises are divided into labour-intensive and capital-intensive enterprises according to the median ratio of capital to labour. In labour-intensive enterprises, EPU has a significant impact on EP, whereas in capital-intensive enterprises, EPU has no significant negative impact on EP (Table 5). Labour-intensive enterprises are mostly light industry, handicraft industry and contract processing enterprises, etc., with low added value of their products. When the EPU increases, enterprises reduce R&D and human capital investment to keep more cash, thus reducing their innovation level, management talent stock and financing ability. Capital-intensive enterprises have relatively abundant capital and fewer financing difficulties. Owing to their advanced production and pollution discharge technologies, the high added value of products, and stable economic benefits, they can obtain more foreign investment. Although foreign investment brings sufficient capital, it also spills out advanced green technology, which lowers the pollution emissions of capital-intensive enterprises [50]. Therefore, the negative effect of EPU on the productivity of labour-intensive enterprises is greater than that of capital-intensive enterprises.

**Table 5:** Heterogeneity analysis of enterprise type and enterprise pollution control intensity.

Variables	labour-intensive enterprises	capital-intensive enterprises	low waste treatment intensity	high waste treatment intention
	ep	ep	ep	ep
ep	-0.160***	-0.016	-0.071***	-0.038***
	(0.023)	(0.010)	(0.014)	(0.011)
Control variables	Yes	Yes	Yes	Yes
Enterprise	Yes	Yes	Yes	Yes
Industry	Yes	Yes	Yes	Yes
Region	Yes	Yes	Yes	Yes
Time	Yes	Yes	Yes	Yes
Obs	8,487	8,448	4,134	15,674
R-squared	0.431	0.454	0.444	0.432

Note: Z-statistics values are reported in parentheses();

*, **, *** denote the significance at 10%, 5%, 1% levels, respectively.

#### Heterogeneity of enterprise pollution control intensity

According to the median number of wastewater and waste gas treatment facilities, enterprises are divided into enterprises with low waste treatment intensity and those with high waste treatment intention. EPU has a greater negative impact on enterprises with low waste management intensity than those with high waste management intensity (Table 5). Owing to its strong waste treatment capacity, the enterprise itself has a small amount of sewage discharge; therefore, the increase in environmental uncertainty has a small impact, and the low emission intensity of enterprises leads to a large amount of pollution discharged by enterprises. When the EPU increases, enterprises must adjust their output to meet the standards of pollution reduction, which leads to idle production equipment and labour force of enterprises, and ultimately reduces the productivity of enterprises [52].

### Robustness test

#### Change explanatory variables

This section remeasures EPU to verify whether its impact on EP is robust. The previous section mainly referred to Ghosh and Olsen (2009) [42] and other algorithms. In this section, the past three years in the previous algorithm are changed to the past five years, and the EPU is calculated according to the same algorithm mentioned above. The regression results shows that the impact of EPU on EP is still significantly negative (Table 6), indicating that the research conclusions of this paper are robust and reliable.

**Table 6:** Change explanatory variable.

Variables	(1)	(2)	(3)	(4)	(5)
	ep	ep	ep	ep	ep
epu	-0.012***	-0.012***	-0.012***	-0.006***	-0.002***
	(0.000)	(0.000)	(0.000)	(0.001)	(0.001)
is		2.109***	2.126***	2.215***	2.039***
		(0.224)	(0.223)	(0.286)	(0.289)
gdzc			0.012***	0.011***	0.012***
			(0.001)	(0.002)	(0.002)
wrpfqd				-0.001***	-0.001***
				(0.000)	(0.000)
zcfzl					-0.180***
					(0.036)
Enterprise	Yes	Yes	Yes	Yes	Yes
Industry	Yes	Yes	Yes	Yes	Yes
Region	Yes	Yes	Yes	Yes	Yes
Time	Yes	Yes	Yes	Yes	Yes
Obs	45,436	45,429	45,418	29,846	28,319
R-squared	0.301	0.303	0.305	0.335	0.370

Note: Z-statistics values are reported in parentheses();

*, **, *** denote the significance at 10%, 5%, 1% levels, respectively.

#### Change explained variable

The explained variable used in the previous section was measured using the LP method. In this section, the semi-parametric OP method is used to measure EP. The negative effect of EPU on EP is still significant(Table 7), indicating that the conclusions of this study are robust and reliable.

**Table 7:** Change explained variable.

Variables	(1)	(2)	(3)	(4)	(5)
	ep	ep	ep	ep	ep
epu	-0.011***	-0.011***	-0.011***	-0.007**	-0.046***
	(0.003)	(0.003)	(0.003)	(0.003)	(0.008)
is		2.076***	2.082***	1.650**	1.557*
		(0.628)	(0.629)	(0.725)	(0.822)
gdzc			0.003	0.003	0.006
			(0.003)	(0.003)	(0.004)
wrpfqd				-0.002**	-0.002**
				(0.001)	(0.001)
zcfzl					-0.131
					(0.119)
Enterprise	Yes	Yes	Yes	Yes	Yes
Industry	Yes	Yes	Yes	Yes	Yes
Region	Yes	Yes	Yes	Yes	Yes
Time	Yes	Yes	Yes	Yes	Yes
Obs	23,268	23,268	23,263	18,094	16,881
R-squared	0.055	0.057	0.057	0.059	0.063

Note: Z-statistics values are reported in parentheses();

*, **, *** denote the significance at 10%, 5%, 1% levels, respectively.

#### Eliminate the sample of entering, exiting enterprises and Winsor

The impact of EPU on EP may be affected by enterprise entry and exit. So this part deletes the sample of newly entering and exiting enterprises during the study period, and Table 8 column (1) shows that the coefficient of EPU is significantly negative, which is consistent with the benchmark regression results.

**Table 8:** Adjustment of sample range and endogeneity tests.

Variables	(1)	(2)	(3)	(4)
	Eliminate entry exit sample	2008–2014	Delete the time dummy variable	Add variable L.EP
	ep	ep	ep	ep
epu	-0.052***	-0.141***	-0.038***	-0.040***
	(0.010)	(0.026)	(0.011)	(0.008)
Control variables	Yes	Yes	Yes	Yes
Enterprise	Yes	Yes	Yes	Yes
Industry	Yes	Yes	Yes	Yes
Time	Yes	Yes	Yes	Yes
Region	Yes	Yes	Yes	Yes
Obs	13,666	6,226	19,064	18,928
R-squared	0.435	0.048	0.069	0.435

Note: Z-statistics values are reported in parentheses();

*, **, *** denote the significance at 10%, 5%, 1% levels, respectively.

#### Change sample period and delete the time dummy variable

In 2008, China introduced environmental protection law. In order to eliminate the impact of this policy, samples from 2008 to 2014 were used for regression, and the results are shown in Table 8 column (2). In addition, this study chooses to further control the influence of time effect by deleting time dummy variables, as shown in Table 8 column (3). The results show that EPU coefficient is significantly negative, it indicating that the benchmark regression conclusion is robust and reliable.

### Endogeneity test

Since EPU is affected by national macro policies, individual micro behaviors of enterprises are difficult to cause fluctuations in EPU, so there is no reverse causality between EP and EPU. In addition, enterprise, industry, region and year are controlled in the benchmark regression, which can effectively avoid the endogeneity problem caused by omitted variables.

In order to eliminate the influence brought by the autocorrelation of EP, the method of Fang et al. (2015) [53] is used for reference to introduce the one-stage lag of the explained variable into the benchmark regression. Table 8 column (4) shows that the significance of EPU has not changed.

### Mechanism analysis

The starting point for analyzing the mechanism of causal action is that a causal relationship may not affect all individuals and all points in the same way, so it is vital to explore how the strength of the causal relationship varies with object features and actual conditions. This type of analysis is commonly referred to as Moderating Effect analysis. This research employs Moderating Effect to investigate the specific routes through which EPU impacts EP in order to determine whether the Mathematical Derivation is true. In order to investigate the effect mechanism of EPU on EP, this study analyzes three aspects: technological innovation, human capital and foreign direct investment. The specific model is as follows:

$$ep_{ijkt}=\beta_{0}+\beta_{1}epu_{ijkt}+\beta_{\text{2}}iq_{ijkt}+\beta_{3}epu_{ijkt}\cdot iq_{ijkt}+\beta_{4}X_{ijkt}+\lambda_{i}+\lambda_{j}+\lambda_{k}+\lambda_{t}+\varepsilon_{ijkt}$$ (22)

$$ep_{ijkt}=\beta_{0}+\beta_{1}epu_{ijkt}+\beta_{\text{2}}rlzb_{ijkt}+\beta_{3}epu_{ijkt}\cdot rlzb_{ijkt}+\beta_{\text{4}}X_{ijkt}+\lambda_{i}+\lambda_{j}+\lambda_{k}+\lambda_{t}+\varepsilon_{ijkt}$$ (23)

$$ep_{ijkt}=\beta_{0}+\beta_{1}epu_{ijkt}+\beta_{\text{2}}fdi_{ijkt}+\beta_{3}epu_{ijkt}\cdot fdi_{ijkt}+\beta_{\text{4}}X_{ijkt}+\lambda_{i}+\lambda_{j}+\lambda_{k}+\lambda_{t}+\varepsilon_{ijkt}$$ (24)

Among them, *iq*, *rlzb* and *fdi* represent the innovation capability, human capital level and foreign direct investment scale of enterprises respectively. The remaining variables are as described above.

#### Enterprise innovation

Table 9 Column (1) shows that the interaction term between EPU and enterprise innovation is significantly negative, indicating that EPU inhibits EP growth by hindering the improvement of enterprise innovation capability.

**Table 9:** Mechanism test results.

Variables	(1)	(2)	(3)
	ep	ep	ep
epu	-0.038***	-0.008	-0.038***
	(0.008)	(0.012)	(0.008)
iq	0.016**		
	(0.007)		
EPU·iq	-0.009*		
	(0.005)		
rlzb		-0.671	
		(2.306)	
EPU·rlzb		-0.897***	
		(0.229)	
fdi			0.016**
			(0.007)
EPU·fdi			-0.009*
			(0.005)
Control variables	Yes	Yes	Yes
Enterprise	Yes	Yes	Yes
Industry	Yes	Yes	Yes
Time	Yes	Yes	Yes
Region	Yes	Yes	Yes
Obs	16,548	16,548	16,548
R-squared	0.433	0.436	0.433

Note: Z-statistics values are reported in parentheses();

*, **, *** denote the significance at 10%, 5%, 1% levels, respectively.

#### Human capital

Table 9 Column (2) shows that the interaction term between EPU and human capital is significantly negative, it means EPU inhibits EP increase by hindering the improvement of enterprise human capital level.

#### Foreign direct investment

Table 9 Column (3) shows that the interaction term between EPU and foreign investment is significantly negative, which indicates that EPU inhibits EP increase by hindering the increase of fdi.

## Discussion

Based on combined data from the CIED, CIEPED, and CPD from 2005 to 2014, we empirically analysed the impacts of EPU on EP.

### Contribution

Our study’s contributions are in several aspects. First, the existing literature on EPU is scattered, and lack of quantitative analysis of EPU. Therefore, this study uses bibliometrics and visual analysis methods to identify the core issues of EPU and provides a useful reference for future research on EPU.

Second, the existing literature mostly discusses the impact of government environmental regulation on EP from a macro perspective, while few studies focus on the impact of environmental policy uncertainty on EP. Therefore, this study uses the matched data of CIED, CPD and CIEPED to investigate the impact of EPU on EP and the mechanism between them, which provides a new research perspective for EP improvement.

Third, this study discusses the heterogeneity of enterprise ownership, enterprise industry category, enterprise age, enterprise size, enterprise type, and enterprise pollution control intensity. Moreover, this study reveals that EPU affects EP through technological innovation, human capital and foreign direct investment, which further deepens the understanding of the complete logical chain of EPU on EP.

### Limitations

There are still some limitations in this study, which need to be further deepened. Since the latest statistical year of CIED, CIEPED, and CPD are 2014, we will further carry out relevant research when the data is updated.

From the aspects of research content, in this study we only explore the influence factors of enterprise innovation, human capital and foreign direct investment, and other potential confounding factors should be explored in depth. From the aspects of research methods, this study only examined the linear relationship between EPU and EP, and future research can explore whether there is an asymmetric relationship between them. such as the regional spillover effects of EPU.

## Conclusion and policy implications

### Conclusion

Based on combined data from the CIED, CIEPED, and CPD from 2005 to 2014, this study first discusses the impact of EPU on EP from theoretical and empirical perspectives. Second, based on the heterogeneity of enterprise ownership, enterprise industry category, enterprise age, enterprise size, enterprise type, and enterprise pollution control intensity, the heterogeneity of EPU on EP is discussed. Finally, we empirically examine whether EPU can hinder EP by inhibiting innovation, human capital, and foreign investment. The results are as follows.

First, EPU significantly inhibits the improvement of EP and has a significant negative impact on the high-quality development of enterprises.

Second, EPU mainly inhibits the improvement of EP through enterprise innovation, human capital, and foreign investment. That is, EPU inhibits the improvement of EP by inhibiting the improvement of enterprise innovation ability and reducing the stock of enterprise human capital and foreign investment.

Finally, the inhibitory effect of EPU on EP is characterized by firm heterogeneity, among which non-state-owned enterprises, enterprises in polluting industries, young enterprises, small and medium-sized enterprises, labor-intensive enterprises and enterprises with low waste management intensity have more significant inhibitory effect.

### Policy enlightenment

The conclusions of this paper are helpful for the government to formulate green and efficient development strategies.

1. Prior to developing and implementing environmental policies, all levels of government should thoroughly absorb historical experiences, actively learn from and widely promote beneficial policies, improve the effectiveness of environmental policies, and avoid oscillations and contradictions caused by poor policy outcomes. To increase the stability of corporate expectations and the rationality of corporate investment decision expectations, the government should improve market information transparency and strengthen timely, effective, and in-depth communication and dialogue between the government and enterprises. The government should maintain the sustainability of local environmental regulation policies and reduce the negative impact of EPU on enterprise development. When environmental policy uncertainty grows, the government should gradually improve the transparency and efficacy of environmental policy formation. For example, when the government’s main leadership changes, the new leadership should continue to strengthen environmental oversight of polluting enterprises, and the government should maintain subsidies for talent introduction, innovative technology research and development. Simultaneously, the government need apply environmental protection pressure on enterprises through administrative regulations, as well as encourage them to cut pollution emissions through technological progress and green innovation.
2. Furthermore, because environmental policy uncertainty primarily inhibits corporate productivity improvement through three channels: enterprise innovation, human capital, and foreign investment, it is prudent to strengthen policy support for innovation by improving market competition mechanisms, restraining industry monopolies, and increasing financial support, as well as to continue to implement the strategy of building a talent-strong nation, improving the quality of human capital, and increasing financial support. In the process of policy formulation and implementation, the government needs to formulate more detailed measures according to different types of enterprises, so as to minimize the negative impact of EPU on enterprise development. Financial subsidies and tax relief should be aggressively provided to non-state-owned enterprises, small and medium-sized enterprises, and young enterprises, and the government should promise to provide minimum support years (such as 10 years, 15 years, etc.) to reduce the negative impact of environmental policy uncertainty on enterprise’s productivity. Polluting industries enterprise and low waste treatment intensity enterprise should continue to increase environmental supervision. In general, this paper provides a new research perspective and micro evidence for the efficient development of Chinese enterprises.

## Supporting information

S1 Data (XLSX)
